# Genomic Medicine and Advances in Vaccine Technology and Development in the Developing and Developed World

**DOI:** 10.3390/vaccines9010009

**Published:** 2020-12-24

**Authors:** Rossella Cianci, Laura Franza

**Affiliations:** 1 Internal Medicine, Università Cattolica del Sacro Cuore, Fondazione Policlinico Universitario A. Gemelli IRCCS, 00168 Roma, Italy; 2 Emergency Medicine, Università Cattolica del Sacro Cuore, Fondazione Policlinico Universitario A. Gemelli IRCCS, 00168 Roma, Italy; cliodnaghfranza@gmail.com

Vaccinations are one of the most effective public health measures available at present. Yet, a growing skepticism surrounding vaccinations is causing a lower vaccination rate than desirable [1,2]. Furthermore, lack of vaccinations leads to a lack of infectious disease control and/or eradication. Vaccines must have low rates of side effects, have to be unexpensive, effective and widely disposable. The new technologies must be made available to quickly develop new vaccines for rapid spreading diseases. During the past decades, several ways have been used to inoculate vaccines: intramuscular, intranasal and oral. The skin too can represent a good site for vaccination, as reviewed in [3]. Moreover, a heavy progress has been made in vaccine development and technologies. In fact, vaccination strategies available at the moment are very different from the ones used in the past, both in terms of targets and techniques behind them [4].

In this Special Issue of *Vaccines*, several authors have examined and discussed new programs and technologies proposed for the development of modern vaccines to prevent infectious diseases.

Vaccinations are important, in particular, in low-income areas of the world, given the few resources available to eventually treat diseases [5]. Polio; hepatitis B (HBV); tick-borne encephalitis [6]; and Venezuelan, Eastern and Western equine encephalitis, for instance, are common in rural areas and present an important economic burden, thus needing an effective vaccination strategy to prevent them [7]. For example, Chinnakannan et al. performed a study in which they used a genetically adjuvanted chimpanzee adenovirus and modified vaccinia Ankara vectored HBV vaccines that resulted in a strong HBV-specific T-cell response [8]. Manukyan et al. performed a multiplex PCR-based neutralization assay to test the antibodies against poliovirus in a short time [9]. Moreover, in these areas, it is often necessary to design easy-to-administer vaccinations (e.g., oral forms). Recently, vaccinations against common pathogens, such as *Escherichia coli*, have been designed in oral formulations to enhance vaccinations in more rural areas. Matias et al. used a nanoparticle formulation that was able to achieve oral immunization in pregnant mice and passive immunity in offspring [10]. Infections in stock and cattle increase the risk of starvation; thus, vaccination programs in animals have been encouraged. For such purpose, Song et al. [11] demonstrated that recombinant Marek’s disease virus (rMDV) with reticuloendotheliosis virus (REV)-LTR (long terminal repeat) shows a good safety profile in target chickens and environment and can be used in the development of a vaccine against MDV. An interesting program, as discussed by Antenucci et al., used outer membrane vesicles to immunize chickens against *Gallibacterium anatis*, which was proven both effective and economically advantageous [12]. New vaccination programs have also been carried out for other avian pathogens such as herpes viruses. Tang et al. [13] experimented with a new recombinant vaccine protecting poultry against three avian viral diseases. Other programs have been proposed for the Newcastle disease virus. Bello et al. performed a study using reverse genetics technology to design a modern vaccine and proved a strong antibody-mediated response protecting chickens against the Newcastle disease virus genotype VII [14,15]. In low-income areas, animal-borne infections are also a serious threat to human health, not only because they impoverish the population, but also because the risk of zoonosis in this context is high [16]. Avian influenza, for instance, is a common example of an animal-borne disease that can have serious consequences on human health and has been the object of intense research: a study by Hoang et al., conducted on chickens, showed that vaccinating them against H5N1 strain of influenza protects them against the Vietnamese avian influenza [17]. *Leishmania* is another dangerous pathogen, both for animals and humans. However, two studies conducted on mice have shown that two types of vaccines are actually effective in eliciting a strong CD4 and CD8 T-cell mediated response and IFN-γ and TNF-α production. Agallou et al. showed that multiepitope vaccines combined with the opportune adjuvants eliciting immune response could be used as vaccines against leishmaniasis [18,19]. Other diseases, such as tularemia, rabies and some forms of encephalitis, also place a heavy burden on national health systems, and vaccination programs offer a reasonable solution. For these pathologies, advances have been made and are discussed in this Special Issue. Li et al. [20] reported that costimulatory factor OX40-ligand (OX40L) could represent an adjuvant to develop an immune response to rabies virus and can be used to construct a valid vaccine for animals. Johnson et al. [21] showed the safety and genetic stability of Venezuelan equine encephalitis virus vaccine in a murine model. Natrajan et al. [22] highlighted the possibility to predict the response to tularemia vaccine.

Toxoplasmosis is another example of an animal-borne disease that can have dangerous consequences on people, particularly in pregnant women, a very delicate population [23]. Zhang et al. conducted a study on mice, highlighting that *Toxoplasma gondii* tyrosine hydroxylase (TgTH) could be highly immunogenic and, thus, be an interesting target antigen for a vaccine [24]. Another possible candidate in the development of a *T. gondii* vaccine is a knock-out strand, without the adenylosuccinate lyase gene, which seems to offer wide protection and should be used in clinical trials in humans this year, as discussed by Wang et al. [25]. Another pathogen that often goes undetected in healthy adults but can be dangerous during pregnancy is cytomegalovirus (CMV); at the moment, there only are vaccinations that offer partial protection, but research aiming at achieving full protection is ongoing. The state of the current situation is extensively reviewed by Gerna et al. [26].

Studies have consistently shown that vaccines are safe and effective, and researchers all over have continued studying ways to improve their effectiveness. Some pathogens, indeed, have been studied for years in attempts to develop effective vaccination strategies, given that therapeutic options are not always available or resolutive. Human immunodeficiency virus (HIV) is a perfect example [27]. For years, an effective vaccine has been searched for, without success. Recently, though, different studies have shed some light on strategies which might help in developing an effective vaccination against this virus, not only through the development of an effectively immunogenic vaccination, as discussed by Calado et al. [28], who used the CRF02_AG-based envelope immunogens and prime-boost immunization strategy, but also by taking advantage of the synergic effect of other vaccinations, as discussed by Matchett et al. [29]. 

Research on vaccination is also trying to improve the technology with which vaccines are administered and designed. The vectors through which vaccines are administered, for instance, have gathered a lot of attention. Modified vaccinia virus Ankara, for instance, was studied by Atukorale et al. in terms of its potential for use in viral-vectored vaccine development [30]. Furthermore, whether it could be safely used in vaccination against the herpes virus was studied: it has proven not only safe but also highly effective, given its capacity to stimulate a complex immune response [31]. Moreover, engineered extracellular vesicles can be used as vectors for vaccination, and it is now clear how they need to be constructed to offer the highest immunogenic effect, as shown by Chiozzini et al. [32]. Another interesting aspect that is coming under increased scrutiny is the interaction between host and vaccine. Studies conducted on zebrafish by Pereiro et al., for instance, have proven that phosphatase and TENsin homolog on chromosome 10 (PTEN) can reduce viral spreading but increase viral replication during viral infections, which could impact the effect of vaccinations [33]. Even more interestingly, recent studies have gained insight into the role of γδT cells, which apparently interact in a virtuous circle with immunoglobulin G (IgG), as shown by de Sousa et al. [34].

Part of the research that is now being conducted on vaccinations also concerns the need to design more effective vaccinations for the most vulnerable populations, such as the elderly [35,36]. For instance, vaccines against *Streptococcus pneumoniae*, a very dangerous pathogen, particularly for those over 65 years of age, are being redesigned to offer the maximum efficacy, given the different responses of elderly persons to vaccination. Amonov et al. studied a cpsE-endA double-mutant strain as a candidate for the engineering of novel live attenuated vaccines [37]. Moreover, the vaccine against the respiratory syncytial virus is being revised through a multiepitope-based subunit vaccine using a reverse vaccinology approach, as discussed by Tahir et al. [38].

Aside from infective diseases, vaccinations can be useful against a variety of other conditions. Most notably, vaccination protocols are being used in oncologic diseases, targeting molecules specific to the tumor. Research on the possible molecular targets is currently advancing, as detailed in the review by Buonaguro et al. [39]. Promising results are being obtained in many forms of cancer, for instance, breast cancer, using dendritic cell vaccine immunotherapy [40]. 

Vaccinations are also being used more and more in immune diseases, and promising results are coming from studies in which specific vaccines target interleukin-17 in mice with systemic lupus erythematosus [41], a disease which currently cannot be cured and which causes a high burden in morbidity and mortality [42]. Type 2 diabetes is also a disease in which the immune system plays a key role. Roesti et al. developed a vaccine against amyloidogenic aggregates in pancreatic islets of mice, and the results were quite interesting: vaccination seemed to delay the onset of hyperglycemia and prevent the disease from progressing [43].

Overall, the importance of vaccinations is clearer than ever in the current situation: the rise of the novel coronavirus has shown us all what a world without vaccinations could look like. The research towards a vaccine against this disease is of extreme importance for everyone in the world and reminds us all of the great value and importance of vaccination protocols. Advances in SARS-CoV-2 vaccine development are discussed by Zhang et al. [44].

Vaccinations are fundamental for global health. The development of vaccinations, not only against infective diseases but also against tumors and immune disorders, is an incredible therapeutic opportunity that has thus far given encouraging results. We believe that this Special Issue is of great interest, as it provides information on the state of the art of vaccination science and could be food for thought for future research.

## References

[1] Pandolfi F., Franza L., Todi L., Carusi V., Centrone M., Buonomo A., Chini R., Newton E., Schiavino D., Nucera E. (2019). The Importance of Complying with Vaccination Protocols in Developed Countries: “Anti-Vax” Hysteria and the Spread of Severe Preventable Diseases. Curr. Med. Chem..

[2] Lemoine C., Thakur A., Krajišnik D., Guyon R., Longet S., Razim A., Górska S., Pantelić I., Ilić T., Nikolić I. (2020). Technological Approaches for Improving Vaccination Compliance and Coverage. Vaccines.

[3] Hettinga J., Carlisle R. (2020). Vaccination into the Dermal Compartment: Techniques, Challenges, and Prospects. Vaccines.

[4] Heaton P.M. (2020). Challenges of Developing Novel Vaccines With Particular Global Health Importance. Front. Immunol..

[5] Hardt K., Bonanni P., King S., Santos-Preciado J.I., El-Hodhod M., Zimet G., Preiss S. (2016). Vaccine strategies: Optimising outcomes. Vaccine.

[6] Kubinski M., Beicht J., Gerlach T., Volz A., Sutter G., Rimmelzwaan G.F. (2020). Tick-Borne Encephalitis Virus: A Quest for Better Vaccines against a Virus on the Rise. Vaccines.

[7] Stromberg Z.R., Fischer W., Bradfute S., Kubicek-Sutherland J.Z., Hraber P.T. (2020). Vaccine Advances against Venezuelan, Eastern, and Western Equine Encephalitis Viruses. Vaccines.

[8] Chinnakannan S., Cargill T.N., Donnison T.A., Ansari M.A., Sebastian S., Ni Lee L., Hutchings C., Klenerman P., Maini M.K., Evans T. (2020). The Design and Development of a Multi-HBV Antigen Encoded in Chimpanzee Adenoviral and Modified Vaccinia Ankara Viral Vectors; A Novel Therapeutic Vaccine Strategy against HBV. Vaccines.

[9] Manukyan H., Petrovskaya S., Chumakov K., Laassri M. (2020). Multiplex PCR-Based Neutralization (MPBN) Assay for Titers Determination of the Three Types of Anti-Poliovirus Neutralizing-Antibodies. Vaccines.

[10] Matías J., Pastor Y., Irache J.M., Gamazo C. (2020). Protective Passive Immunity in Escherichia coli ETEC-Challenged Neonatal Mice Conferred by Orally Immunized Dams with Nanoparticles Containing Homologous Outer Membrane Vesicles. Vaccines.

[11] Song C., Yang Y., Hu J., Yu S., Sun Y., Qiu X., Tan L., Meng C., Liao Y., Liu W. (2020). Safety and Efficacy Evaluation of Recombinant Marek’s Disease Virus with REV-LTR. Vaccines.

[12] Antenucci F., Arak H., Gao J., Allahgadry T., Thøfner I., Bojesen A.M. (2020). Hydrostatic Filtration Enables Large-Scale Production of Outer Membrane Vesicles That Effectively Protect Chickens against Gallibacterium anatis. Vaccines.

[13] Tang N., Zhang Y., Sadigh Y., Moffat K., Shen Z., Nair V., Yao Y. (2020). Generation of A Triple Insert Live Avian Herpesvirus Vectored Vaccine Using CRISPR/Cas9-Based Gene Editing. Vaccines.

[14] Bello M.B., Mahamud S.N.A., Yusoff K.M., Ideris A., Hair-Bejo M., Peeters B., Omar A.R. (2020). Development of an Effective and Stable Genotype-Matched Live Attenuated Newcastle Disease Virus Vaccine Based on a Novel Naturally Recombinant Malaysian Isolate Using Reverse Genetics. Vaccines.

[15] Farnós O., Gelaye E., Trabelsi K., Bernier A., Subramani K., Kallel H., Yami M., Kamen A. (2020). Establishing a Robust Manufacturing Platform for Recombinant Veterinary Vaccines: An Adenovirus-Vector Vaccine to Control Newcastle Disease Virus Infections of Poultry in Sub-Saharan Africa. Vaccines.

[16] Graham B.S., Sullivan N.J. (2017). Emerging viral diseases from a vaccinology perspective: Preparing for the next pandemic. Nat. Immunol..

[17] Hoang H.T.T., Nguyen C.H., Nguyen N.T.T., Pham A.D., Nguyen H.T.T., Hoa L.T., Hanh T.X., Chu H.H., Nguyen N.T. (2020). Immunization with the H5N1 Recombinant Vaccine Candidate Induces High Protection in Chickens against Vietnamese Highly Pathogenic Avian Influenza Virus Strains. Vaccines.

[18] Agallou M., Margaroni M., Kotsakis S.D., Karagouni E. (2020). A Canine-Directed Chimeric Multi-Epitope Vaccine Induced Protective Immune Responses in BALB/c Mice Infected with *Leishmania infantum*. Vaccines.

[19] De Brito R.C.F., Ruiz J.C., Cardoso J.M.D.O., Ostolin T.L.V.D.P., Reis L.E.S., Mathias F.A.S., Aguiar-Soares R.D.D.O., Roatt B.M., Corrêa-Oliveira R., Resende D.D.M. (2020). Chimeric Vaccines Designed by Immunoinformatics-Activated Polyfunctional and Memory T Cells That Trigger Protection against Experimental Visceral Leishmaniasis. Vaccines.

[20] Li Y., Zhao L., Sui B., Luo Z., Zhang Y., Wang Y. (2020). Recombinant Rabies Virus Overexpressing OX40-Ligand Enhances Humoral Immune Responses by Increasing T Follicular Helper Cells and Germinal Center B Cells. Vaccines.

[21] Johnson D.M., Sokoloski K.J., Jokinen J.D., Pfeffer T.L., Chu Y.-K., Adcock R.S., Chung D., Tretyakova I., Pushko P., Lukashevich I.S. (2020). Advanced Safety and Genetic Stability in Mice of a Novel DNA-Launched Venezuelan Equine Encephalitis Virus Vaccine with Rearranged Structural Genes. Vaccines.

[22] Natrajan M.S., Rouphael N., Lai L., Kazmin D., Jensen T.L., Weiss D.S., Ibegbu C., Sztein M.B., Hooper W.F., Hill H. (2019). Systems Vaccinology for a Live Attenuated Tularemia Vaccine Reveals Unique Transcriptional Signatures That Predict Humoral and Cellular Immune Responses. Vaccines.

[23] Manjunathachar H., Singh K.N., Chouksey V., Kumar R., Sharma R.K., Barde P.V. (2020). Prevalence of Torch Infections and Its Associated Poor Outcome in High-Risk Pregnant Women of Central India: Time to Think for Prevention Strategies. Indian J. Med. Microbiol..

[24] Zhang Z., Li Y., Li H., Song X., Ma Z., Lu H., Liu S., Zhao Y., Tan M., Wang S. (2020). Identification of Toxoplasma Gondii Tyrosine Hydroxylase (TH) Activity and Molecular Immunoprotection against Toxoplasmosis. Vaccines.

[25] Wang L., Tang D., Yang C., Yang J., Fang R. (2020). *Toxoplasma gondii* ADSL Knockout Provides Excellent Immune Protection against a Variety of Strains. Vaccines.

[26] Gerna G., Lilleri D. (2020). Human Cytomegalovirus Congenital (cCMV) Infection Following Primary and Nonprimary Maternal Infection: Perspectives of Prevention through Vaccine Development. Vaccines.

[27] Burton D.R. (2019). Advancing an HIV vaccine; advancing vaccinology. Nat. Rev. Immunol..

[28] Calado R., Duarte J., Borrego P., Marcelino J.M., Bártolo I., Martin F., Figueiredo I., Almeida S.C.D.P.E., Graca L., Vítor J. (2020). A Prime-Boost Immunization Strategy with Vaccinia Virus Expressing Novel gp120 Envelope Glycoprotein from a CRF02_AG Isolate Elicits Cross-Clade Tier 2 HIV-1 Neutralizing Antibodies. Vaccines.

[29] Matchett W.E., Malewana G.B.R., Mudrick H., Medlyn M.J., Barry M.A. (2020). Genetic Adjuvants in Replicating Single-Cycle Adenovirus Vectors Amplify Systemic and Mucosal Immune Responses against HIV-1 Envelope. Vaccines.

[30] Atukorale V.N., Weir J.P., Meseda C.A. (2020). Stability of the HSV-2 US-6 Gene in the del II, del III, CP77, and I8R-G1L Sites in Modified Vaccinia Virus Ankara After Serial Passage of Recombinant Vectors in Cells. Vaccines.

[31] Anderson E., Lai L., Wrammert J., Kabbani S., Xu Y., Priyamvada L., Hill H., Goll J.B., Jensen T.L., Kao C.M. (2020). Plasmablast, Memory B Cell, CD4+ T Cell, and Circulating Follicular Helper T Cell Responses to a Non-Replicating Modified Vaccinia Ankara Vaccine. Vaccines.

[32] Chiozzini C., Manfredi F., Arenaccio C., Ferrantelli F., Leone P., Federico M. (2020). N-Terminal Fatty Acids of NEFMUT Are Required for the CD8+ T-Cell Immunogenicity of In Vivo Engineered Extracellular Vesicles. Vaccines.

[33] Pereiro P., Figueras A., Novoa B. (2020). Zebrafish pten Genes Play Relevant but Distinct Roles in Antiviral Immunity. Vaccines.

[34] De Sousa T.R., Victor J.R. (2020). Natural Self-Ligand Gamma Delta T Cell Receptors (γδTCRs) Insight: The Potential of Induced IgG. Vaccines.

[35] Villani E., Colloca G., Valente S., Bernabei R. (2017). Vaccination among the elderly: European state of art and the need for a culture shift. J. Gerontol. Geriatr..

[36] Cianci R., Franza L., Massaro M.G., Borriello R., De Vito F., Gambassi G. (2020). The Interplay between Immunosenescence and Microbiota in the Efficacy of Vaccines. Vaccines.

[37] Amonov M., Simbak N., Hassan W.M.R.W., Ismail S., Rahman N.I.A., Clarke S., Yeo C.C. (2020). Disruption of the cpsE and endA Genes Attenuates Streptococcus pneumoniae Virulence: Towards the Development of a Live Attenuated Vaccine Candidate. Vaccines.

[38] Qamar M.T.U., Shokat Z., Muneer I., Ashfaq U.A., Javed H., Anwar F., Bari A., Zahid B., Saari N. (2020). Multiepitope-Based Subunit Vaccine Design and Evaluation against Respiratory Syncytial Virus Using Reverse Vaccinology Approach. Vaccines.

[39] Buonaguro L., Tagliamonte M. (2020). Selecting Target Antigens for Cancer Vaccine Development. Vaccines.

[40] Hafid S.R.A., Radhakrishnan A. (2019). Palm Tocotrienol-Adjuvanted Dendritic Cells Decrease Expression of the SATB1 Gene in Murine Breast Cancer Cells and Tissues. Vaccines.

[41] Koriyama H., Ikeda Y., Nakagami H., Shimamura M., Yoshida S., Rakugi H., Morishita R. (2020). Development of an IL-17A DNA Vaccine to Treat Systemic Lupus Erythematosus in Mice. Vaccines.

[42] Stojan G., Petri M. (2018). Epidemiology of systemic lupus erythematosus. Curr. Opin. Rheumatol..

[43] Roesti E.S., Boyle C.N., Meier D.T., Sande-Melon M., Storni F., Cabral-Miranda G., Knuth A., Lutz T.A., Vogel M., Bachmann M.F. (2020). Vaccination Against Amyloidogenic Aggregates in Pancreatic Islets Prevents Development of Type 2 Diabetes Mellitus. Vaccines.

[44] Zhang J., Zeng H., Gu J., Li H., Zheng L., Zou Q. (2020). Progress and Prospects on Vaccine Development against SARS-CoV-2. Vaccines.

